# A Study From a Psychological Perspective of High Performance to Explore the Relationship Among Resource Bricolage, Social Capital, Entrepreneurial Attitude, and Intention

**DOI:** 10.3389/fpsyg.2022.944151

**Published:** 2022-07-22

**Authors:** Yu-Hsi Yuan

**Affiliations:** Department of Labor and Human Resources, College of Social Science, Chinese Culture University, Taipei City, Taiwan

**Keywords:** entrepreneurial intention, moderating effect, resource bricolage, social capital, entrepreneurial attitude, psychology of high performance, human resources

## Abstract

COVID-19 caused a serious increase in competition due to limited resources. Obviously, it influenced the entrepreneurs' motivation. The entrepreneurial intention, social capital, and resource bricolage ability of individuals were important issues. Thus, exploring an individual's mindset from a psychological perspective of high performance was the advanced issue to deal with social capital promotion. This study developed an instrument adapted from related scales that consisted of 27 items and four factors: social capital, entrepreneurial attitude, resource bricolage, and entrepreneurial intention. The data was collected through an online survey in China and Taiwan by purposive sampling. A total of 692 valid samples provided data for the statistical process. A multiple regression technique was employed in the data process. The instrument passed the validity and reliability test. Data analysis results showed that social capital can positively predict entrepreneurial attitude and entrepreneurial intention. Furthermore, entrepreneurial attitude will affect entrepreneurial intention dramatically. Yet, resource bricolage ability has no moderating effect on social capital and entrepreneurial intention. In addition, resource bricolage ability plays a moderating role between entrepreneurial attitude and entrepreneurial intention. Moreover, it was found that participants demonstrated a high entrepreneurial intention when there was a high entrepreneurial attitude with high resource bricolage ability when the moderating effect was examined. In this study, some practical suggestions are provided for researchers, educators, and entrepreneurs.

## Introduction

The competitive situation and resource contest became more serious in the global industry ecosystem since the COVID-19 pandemic. Hence, people were forced to change their working style from the physical workplace to a virtual online working environment, as the human resources were restructured. Certainly, many people lost their jobs and even their lives because of the pandemic (VanderWeele, 2020; von Wachter, 2020). Entrepreneurship is an important power in triggering social and economic development and the new direction of human resource development. Thus, how cultivating entrepreneurial talents was a vital topic. The perspective of classical economics was to treat land, labor, and capital as the basic input resources of a country's economy (Foley, 2003). But the labor quality of each nation was not equal, which meant not every citizen could contribute their effort to create prosperity for the nation. For example, the innovators could improve production equipment and processes, but their creation required investment by entrepreneurs and capitalists. Moreover, they utilize it for the practical development of the country's economy. Drucker (2014) agreed that innovation and entrepreneurship are the major triggers of current organizations, economic systems, and society. They clearly demonstrated the close connections among innovation, entrepreneurship, and the country's economics.

An individual can improve existing social capital by ethically managing social relations (Spence and Schmidpeter, 2003). From the viewpoint of enterprise, social capital will help firms gain capital and financial assistance. Furthermore, social capital can facilitate organizational learning, e-word-of-mouth marketing, strategic alliance, mergers, and acquisitions, etc. From the individual's point of view, social capital will ease employment transitions leading to a better salary, increase their influence, and success in starting a new business (Liu, 2018; Zhang et al., 2020). In addition, Sweet et al. (2018) found social capital would affect an individual's life quality as well, for instance, happiness, health, long life, etc. It is clear that social capital will affect startups and the success of the entrepreneur (Bizri, 2017). The establishment of a strong social network by entrepreneurs is not without its costs. The social network relies on common activities by connecting multiple stakeholders with different backgrounds. Then social network transforms into social capital and supports the entrepreneur's success (Williams et al., 2017), which is supported by the closer connection between social capital and entrepreneurship.

The entrepreneur begins their startups with limited resources and tries their best to search for resources. Thus, they use the strategy of resource bricolage for problem-solving to survive and develop thereafter (Digan et al., 2019). The concept of resource bricolage includes searching for capital, workforce, skills, and knowledge. It depends on the needs of the entrepreneur. They will maintain their business operation by collecting or composing demand resources. Meanwhile, social capital consisting of social networks is a critical factor for startup resources (Digan et al., 2019; Chen et al., 2020). This highlights how the promotion of social capital will promote an entrepreneur's resource bricolage ability.

Individuals' behavior is significantly affected by their attitudes and intention. They are powerful indicators to predict people's behavior within the framework of the Theory of Planned Behavior (Ajzen and Fishbein, 2000). Entrepreneurial attitude and intention will motivate an individual's entrepreneurial behavior. It is an important step to start a business initially. Social capital can support entrepreneurial attitude and intention as contextual factors (Mahfud et al., 2020). From the perspective of human resource development, an individual's career path can be widened with many options. The most challenging chance for an individual is to be free from financial constraints and be able to put their own ideas into practice (Nabi et al., 2021). However, most studies discussed entrepreneurial attitude, intention, resource bricolage, and social capital from the aspect of finance and management instead of digital human resource development to discover the relationship among these factors. That is why, this study was trying to explore the association among entrepreneurial attitude, intention, resource bricolage, and social capital.

One of the tasks of higher education is to solve economic problems *via* knowledge (Nabi et al., 2010). Faced with the pandemic, governments implemented entrepreneurial policies to maintain pre-pandemic employment levels (Karimi et al., 2017). At the same time, this topic concerned academia and industry. The government also promoted entrepreneurial education (Henry and Lewis, 2018). Entrepreneurial education courses were not only provided in business schools but they were also welcomed in the engineering, social science, and art fields as well (Sun et al., 2017). An important step before supporting entrepreneurial education is to recognize that “entrepreneurship can be taught and learned” (Fretschner and Weber, 2013). Hence, it can cultivate undergraduates' entrepreneurial attitudes and intentions. Entrepreneurial education will systematically train and motivate them to create startups (Pittaway and Cope, 2007; Nabi et al., 2010). Thus, the planning for entrepreneurial education and skill training is an important stage for promoting and motivating an individual's entrepreneurial attitude and intention. It will increase the success of startups, and enhance the country's economic and social development. Furthermore, the current research advantage direction was focused on the psychology of high performance because the psychosocial skill was the new research gap for the talents' development (Olszewski-Kubilius et al., 2019). Therefore, it tries to explore the associated factors of social capital promotion strategy *via* the psychology of the high-performance perspective of the researcher.

Meanwhile, social capital included digital human resource development, and this study explored the mindset of an individual's resource bricolage, social capital, entrepreneurial attitude, and intention. These factors will reflect an individual's ability toward entrepreneurship through their social capital. Furthermore, under the information and communication technology (ICT) environment, these subjects will present their digital human resource development potential to support their career competency based on the psychological perspective of high performance.

This study sought to provide a perspective on digital human resource development in which the association among entrepreneurial attitude, intention, resource bricolage ability, and social capital was studied. Before this, there were insufficient studies discussing the moderating effect of resource bricolage ability between entrepreneurial attitude and intention. The results of this study will influence the government's education policy and reinforce entrepreneurial opportunities and success in practice. That resource bricolage ability played a moderating role in the research framework and also filled in a research gap, offering practical value for both academia and industry.

## Literature Review

### Social Capital

Bourdieu (1986) has shown that the meaning of social capital is an integration of substance resources and potential resources. A high level of social capital is connected with a sustainable and stable social network. A stable social network is established through formal relationships that are recognized by all members accepted into the system. An individual's social capital was defined as a social network scope that is supported by members' integrated capitals (Bourdieu, 1986). Engbers et al. (2017) agreed with a definition of social capital that focuses on the functional dimension. Social capital functions to link large numbers of people who have the same characteristics. It consists of social structures that align resources to assist members in the network to reach their goals. Social capital is similar to other types of capital in that productivity is increased. Indeed, social capital is capable of contributing to the achievement of expected goals as other types of capital do (Engbers et al., 2017). Social capital originated from the idea of social status that presented useful concrete and potential resources. The challenge is whether an individual can use this power to achieve their aims effectively. Thus, social status is treated as social capital. This phenomenon demonstrates social capital as practical assistance to a person (Chen and Li, 2017). Social capital is a highly productive force that increases the possibility of fulfilling expectations (Hernández-Carrión et al., 2017). Social capital provides rich social relationships, the chance to improve social skills, social network access, and enhanced interpersonal communication (Bradford et al., 2019). Benbow and Lee (2019) believed that social capital is interpersonal connections or social networks, with norms and trusts integrated into it. Furthermore, social networks and trust have a mutual relationship that reinforces one another (Chen et al., 2018). Social capital has a bridging-building function in nature that can connect various levels of society. It was found that social capital would stimulate the accumulation of resources and encourage individuals to pursue entrepreneurial careers, and others to go into the field of entrepreneurial research (Davidsson et al., 2017; Grzegorczyk, 2019). Social capital also supports the mental health of entrepreneurs (Shepherd and Krueger, 2002), and it will encourage individuals to directly commit to starting a new business (Wang et al., 2019). Consequently, social capital acts as a trigger to begin an entrepreneurial career, contributing to their survival, and their development during the resource gain. When an individual has enough social capital and direct entrepreneurial experience, awareness of their own ability will be enhanced, and directly affect their entrepreneurial attitude (Mahfud et al., 2020). Hence, having rich social capital will affect an entrepreneur's attitude and intention positively and play an important role in those factors.

### Digital Human Resource Development

Following the development of information and communication technology, the length of time between generations of technology had been shortened, and the knowledge base to utilize the technology has changed just as rapidly. Emerging digital technology has brought about a lifestyle of convenience and deeply affected working styles and approaches to work in general, particularly impacting the digitalized human resource development field (Adam et al., 2020). Management in traditional enterprises and organizations was based on a departmental structure and the job positions within it. Now in the digital era, people and tasks have been the central focus rather than strict adherence to traditional managerial structures under a trend that has crossed the boundaries of various industrial fields (Williams and Schubert, 2018). Project teams are organized in a manner similar to “a bunch of grapes,” hence replacing the hierarchical structure that resembled a pyramid. The replacement of the traditional job-centered organization has indicated a new direction in digital human resource development (Lee, 2021). Therefore, different competencies and working styles will be required of employee talent, and job titles in an organization will reflect this: Digital Transformation Managers, Scrum Masters, Scrum Development Coaches, and Quality Research and Development Engineers (Bibik, 2018). These will become competencies that increase the demand for modern manpower that focus on digital management skills and the ability to apply digital technology (Thite, 2020). Moreover, those competencies require flexibility to allow their application across industries and occupations, and digital human resource development needs to consider this.

Competency consists of digital skills that include digital technique, data analysis, digital design, digital media, information and communication technology, and different digital systems. Optimized Scrum thinking skills needed to deal with a complicated and uncertain digital world. This complexity poses a barrier that will challenge people's comprehension and communication skills across various fields, industries, and businesses. Emerging digital technology has also eliminated geographical boundaries. Hence, individuals should be equipped with the ability to operate in an international environment. Thus, competency includes the ability to manage a multinational staff in an international market, communicate in multiple languages, and adapt to different cultures (Rahimi et al., 2021). The described competencies have become essential requirements for entrepreneurs who live in the digital age. Consequently, it is of significance to cultivate these competencies in entrepreneurs through the field of human resource development. This study applied this theoretical perspective of human resource development to construct an entrepreneur's cultivation structure.

### The Psychology of High Performance

It concerned an individual's development process from childhood to the adult through the psychology of high performance, especially the development from potential to creative achievement (Olszewski-Kubilius et al., 2019). The individual's competency to be cultivated in a long term from the stage of infancy, childhood, youth, adult, mature, and old age reflected the different degrees of abilities (Subotnik et al., 2018). Subotnik et al. (2019) pointed out that talent development was the process from “being” to “doing” of the adult's domain knowledge. Moreover, the contents of adult domain knowledge including intrinsic and extrinsic knowledge, correlated with creativity and entrepreneurship significantly (Siachou et al., 2021). Indeed, the main strain of the psychology of high-performance study was concentrated in discover the path of the excellent performance of talents or entrepreneurs (Subotnik et al., 2018). It identified that the talents were unequipped by gifts. Instead, scholars agreed that a marked high-performance talent relies on the interaction of living environment, learning, and experience accordantly. The talents experienced heavy challenges, frustrations, and dangerous hindrances in their life (Olszewski-Kubilius and Thomson, 2015; Subotnik et al., 2019). This argument suits for outlines the mental process of entrepreneurs. Studies provided evidence to support the relationships between entrepreneurial attitude and intention due to the strongest psychological quality that will enhance entrepreneurs' attitudes and intentions. Thus, the entrepreneurs' performance will be promoted dramatically (Sivarajah and Achchuthan, 2013). Moreover, Anwar and Clauß (2021) argued that the top managers' psychological factors can result in high performance in bricolage and problem-solving. Hence, it provided an obvious connection between the psychological perspective of high performance and an individual's entrepreneurial attitude, intention, and bricolage ability.

According to the perspective of the psychology of high performance, the mental model and quality of entrepreneurs and their behaviors were highly based on this theory. Thus, it becomes the foundation theoretical base of this study.

### Entrepreneurial Attitude and Intention

Robbins (2001) emphasized that an individual's attitude will impact his or her behavior directly. He thought that attitude was an antecedent before acting, affecting behavioral intention. Scholars have provided a critical factor necessary to predict an individual's behavior (Fishbein and Ajzen, 1975; Ajzen, 1991). It has been demonstrated that behavioral intention increases as an individual's attitude increases. On the contrary, an individual's deeper negative attitude will cause lower behavioral intention. Therefore, it is a valid approach to measure people's behavioral intention as a function of attitude. From the perspective of entrepreneurial research, the entrepreneurial attitude was an individual's antecedent signal related to the success or failure of a startup (Zhao et al., 2005). Fitzsimmons and Douglas (2011) believed that a passionate entrepreneur has a proactive attitude toward a startup, and it will support an entrepreneur's confidence when overcoming challenges during the process of starting a new business. Moreover, the passion will transfer to positive emotion toward the entrepreneurial attitude (Mageau et al., 2009). Certainly, positive emotion and entrepreneurial attitude will drive entrepreneurial works in practice (Cardon et al., 2009). Consequently, a person with a high entrepreneurial attitude will affect his or her entrepreneurial intention (Hornsey and Fielding, 2017; Turner and Gianiodis, 2018).

**Figure 1:**
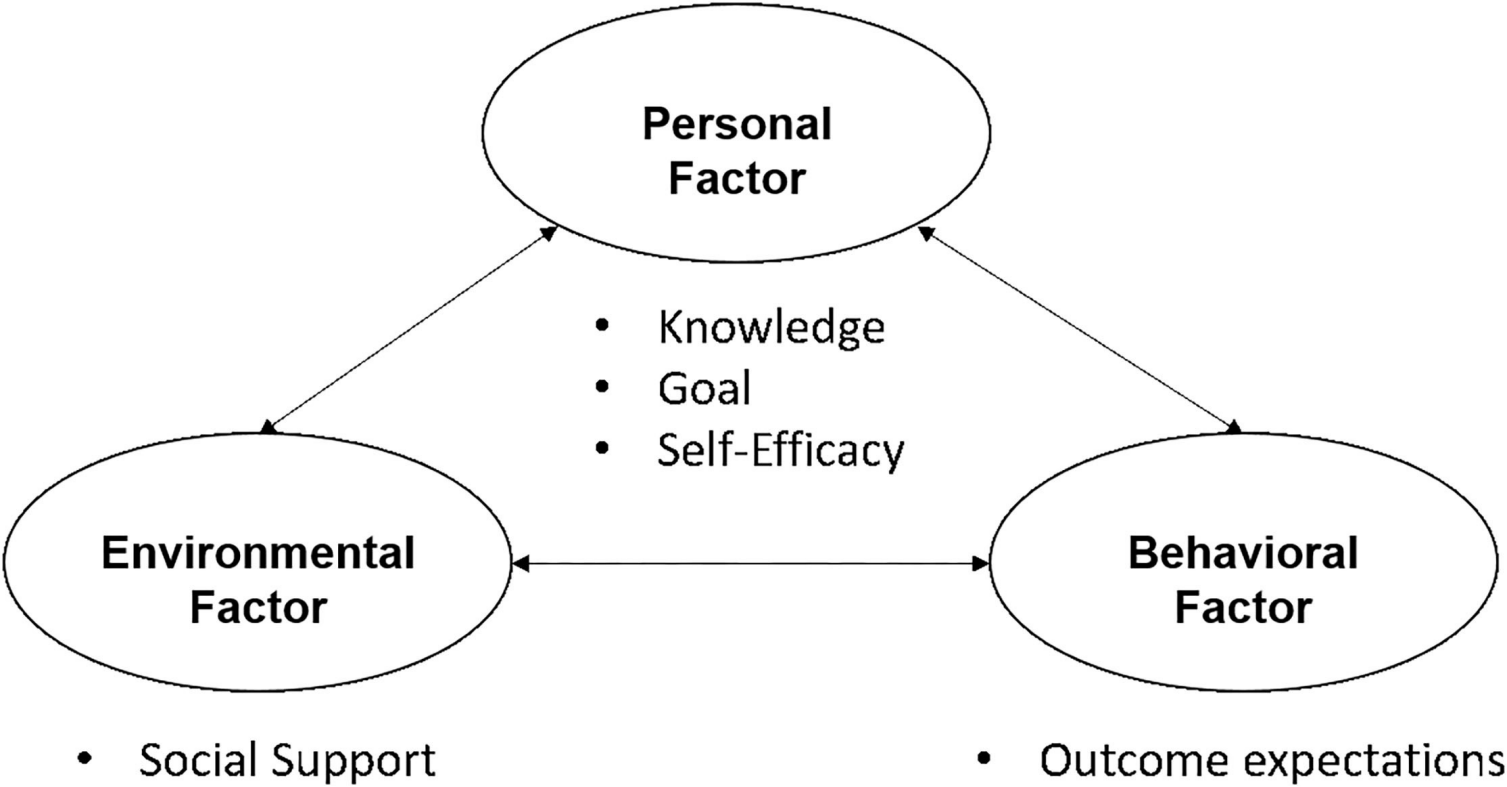
The social cognitive theory and the interaction factors. Source: Schunk and DiBenedetto (2020).

Bonesso et al. (2018) agreed attitude contains three parts. These are cognition, emotion, and behavioral intention. These are reflected in an entrepreneurial attitude as well (Bonesso et al., 2018): (1) cognition—the inner perception of entrepreneurial attitude, (2) emotion—the degree of preference for entrepreneurship, and (3) behavior and intention—the tendency to engage in entrepreneurial behavior. Bandura (1986) put forward the theory of social cognition (see Figure 1). He believed that personal, environmental, and behavioral factors influence each other. Furthermore, corresponding actions are gradually produced in this process. As for the personal factor, focusing on entrepreneurship, attitude is the basis and conscious process for future entrepreneurial behavior.

Hence, social cognition has shown that behavioral tendencies are a major part of the composition of attitudes. In fact, a strong entrepreneurial intention is a vital condition for creating behavior leading to a startup (Weiss et al., 2019). An individual's intention, experience, and mental state will guide his or her critical thinking and decision-making (Butler et al., 2017). It can be seen from this that entrepreneurial intention is necessary for successful entrepreneurship. Specific to behavior, Krueger (2000) believed that entrepreneurial intention is the preparatory behavior for entrepreneurial action and the implementation of startups. Ridha and Wahyu (2017) defined it more broadly not only as a specific behavior or preparation to act but also as a performance of the act. To sum up, social capital will affect an individual's cognition and behavior significantly, and a person's entrepreneurial attitude demonstrates whether the judgment toward entrepreneurial behavior is positive or negative (Mahfud et al., 2020). Lan and Luc (2020) agreed that a person who has decided to start a new business has entrepreneurial intention ahead of entrepreneurial behavior, and it will be directly affected by entrepreneurial attitude. Therefore, this study used social capital as the independent variable to predict various dependent variables:

*Hypothesis 1: Social capital can predict an individual's entrepreneurial intention positively*.
*Hypothesis 2: Social capital can predict an individual's entrepreneurial attitude positively*.
*Hypothesis 3: Entrepreneurial attitude can predict an individual's entrepreneurial intention positively*.

### Resource Bricolage Ability

“Bricolage” is a loanword in English from the French, basically meaning “do-it-yourself.” It is defined by Merriam-Webster (2021) as using whatever comes to hand to construct something. Mzembe et al. (2019) referenced Lévi-Strauss (2004) definition of bricolage that he used in the book “The Savage Mind,” meaning people who use whatever is at hand instead of “looking for predetermined resources for his/her projects as a bricoleur” (Mzembe et al. 2019, p. 997). Moreover, Mzembe et al. (2019) based on their conception of bricolage on Lévi-Strauss' definition and identified its contents as being: (1) a reserve of resources to be acted upon or exploited; (2) a process of functional integration of these resources; and (3) output of the combined resources. Since the early twentieth century, bricolage has been closely associated with entrepreneurship (Baker and Nelson, 2005; Mzembe et al., 2019). Baker et al. (2003) defined bricolage as the integration of local resources to solve problems and create new opportunities. For an entrepreneur, the resources available might not be immediately suitable for the problem at hand, but they are repurposed to meet the challenges of a changing business environment. This active retooling of local resources is bricolage (Baker and Nelson, 2005). A power driving startup is the ability to collect and integrate local resources and then recombine them for new purposes. Daily business operations compound the pressure created by the need to reutilize resources as there are time limits that need to be met. An entrepreneur must expend a great deal of effort to use incomplete tools or materials for useful purposive solutions for startups (Mzembe et al., 2019). Bricolage is not only the recombination of physical resources but also a review of intellectual and organizational resources and recombining them into means for solving problems. Entrepreneurs can redefine physical and non-physical materials with different attributes and combine them into new values to solve existing problems (An et al., 2018). In the process of making do, messages or signals will appear giving the entrepreneur information about the suitability of the recombination of resources (Di Domenico et al., 2010). In summary, the ability of entrepreneurs to bricolage resources can be regarded as the ability to repurpose resources whether they are physical, intellectual, or organizational to overcome the difficulties faced by them with regard to starting a business. The ability to adjust to change in the form of the bricolage is important to cultivating entrepreneurship. Related studies support the idea that social capital can predict “entrepreneurial intention” and “resource bricolage ability” (Chen et al., 2020; Mohammadi, 2021). Therefore, the research hypothesis is as follows:

*Hypothesis 4: An individual's resource bricolage ability plays a moderating role between social capital and entrepreneurial intention*.

Of course, the relationship between entrepreneurial attitude and entrepreneurial intention is supported by academic research (Do and Dadvari, 2017; Kusmintarti et al., 2017; Mahfud et al., 2020). However, insufficient studies have been done to verify the associations between these two and resource bricolage ability. Therefore, the hypothesis emerged as follows:

*Hypothesis 5: An individual's resource bricolage ability plays the role of moderator between entrepreneurial attitude and entrepreneurial intention*.

## Research Design

### Framework

**Figure 2:**
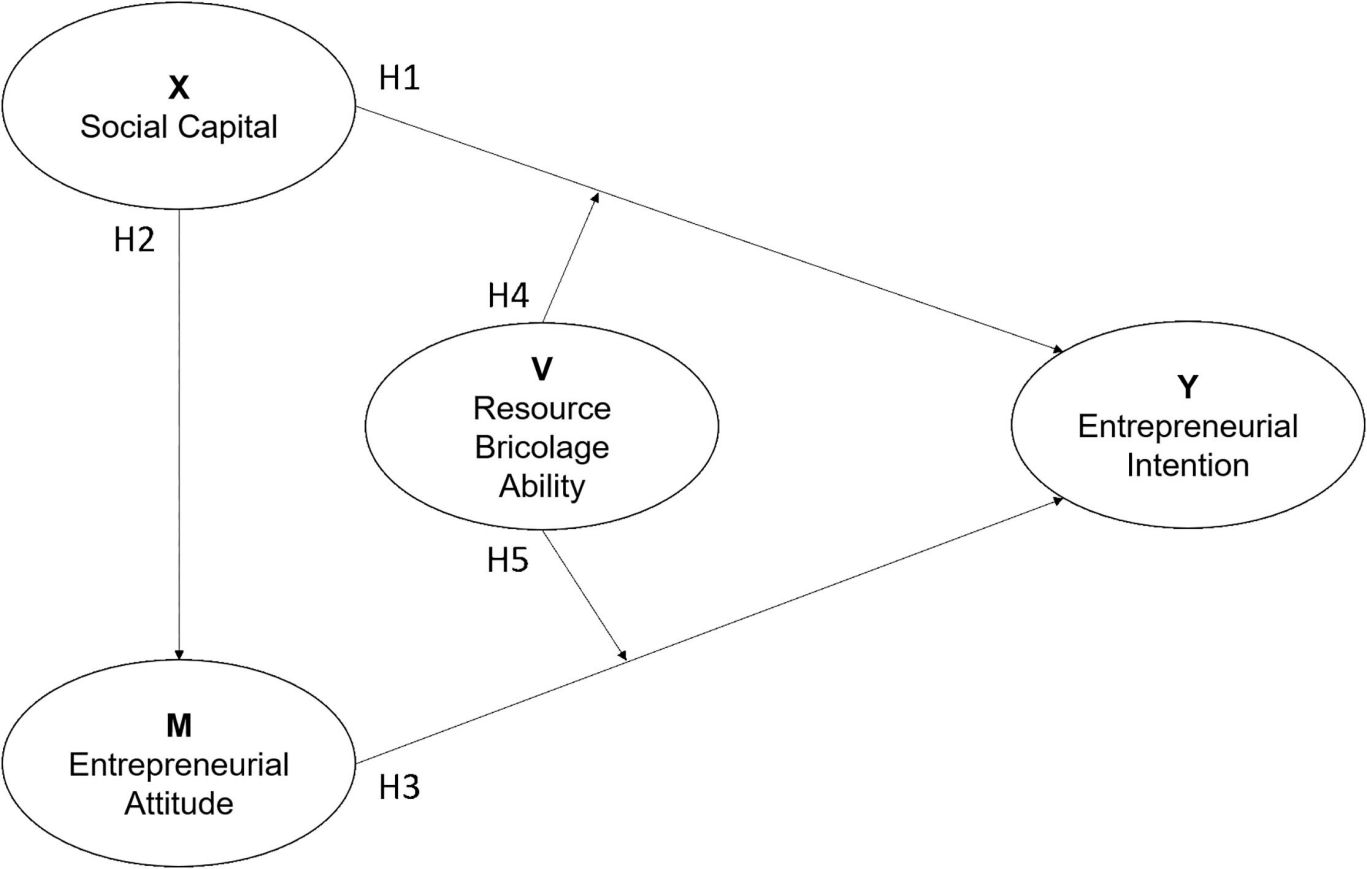
The framework of the study.

To explore the research issue, this study provides a proposed framework in Figure 2, which is based on the literature and the researcher's perspective. It assumed the independent variable “social capital” (X) would affect the dependent variable “entrepreneurial attitude” (M) and “entrepreneurial intention” (Y), respectively. Furthermore, the “entrepreneurial attitude” (M) will be an independent and mediating variable in this model to predict the “entrepreneurial intention” (Y). Meanwhile, “resource bricolage ability” (V) will impact the relationship between “social capital” (X) and “entrepreneurial intention” (Y). It will also have a moderating effect on “entrepreneurial attitude” (M) and “entrepreneurial intention” (Y). This follows Hayes (2018) perspective, as he calculated V to Y, XV to Y, and VM to Y.

### The Respondents

The survey subjects were invited to answer the online questionnaire designed by the researchers through purposive sampling (Etikan et al., 2016). The subject selection criteria focused on joined entrepreneurship training or related activities at universities or training institutes in Mainland China and Taiwan. Those subjects are serving as managers, executives, or owners of enterprises. A total of 1,200 survey questionnaires were distributed in 2021 to the participants *via* trainers, lecturers, seminar sponsors, or related activities organizers. At least, there were 692 respondents as survey data for the further statistical analysis process.

### The Survey Instrument

The survey items were collected from related studies that included a questionnaire. All of the survey items are summarized in Table 1. Furthermore, the back translation process (Beaton et al., 2000; Slavec and Drnovšek, 2012) was employed to comply with the original meaning of the questions referred to in this study.

**Table 1:** The Items and dimensions of the developed questionnaire.

**Factors**	**Dimensions**	**Items**	**Sample items**	**Original source**
Entrepreneurial intention	Proactivity	3	I take the initiative to design entrepreneurial ideas to cope with external environment changes rather than being constrained by it.	Rank and Strenge, 2018
	Risk taking	3	I should try boldly to deal with external changing environment.	
	Innovativeness	3	I care about RandD in my entrepreneurial vision.	
Social capital	–	5	I developing best solutions relay on good relationships with clients, suppliers, and partners.	Subramaniam and Youndt, 2005
Resource bricolage	–	8	I can find solutions to cope new challenges by using existing resources.	Davidsson et al., 2017
Entrepreneurial attitude	–	5	Becoming an entrepreneur was my first choice.	Liñán and Chen, 2009

*Source: By author*.

### Analysis Methods

**Figure 3:**
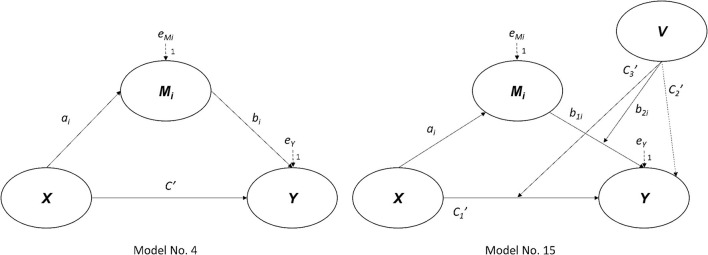
The applied regression analysis model. Source: Referred from Hayes (2017).

A multiple regression analysis was employed for processing the data collected by this research. Furthermore, the regression analysis Model No. 4 (Hayes, 2017) was used to test the mediating effect of Y (entrepreneurial attitude) between X (social capital) and Y (entrepreneurial intention). Model No. 15 (Hayes, 2017) was also applied to verify the moderating effect of V (resource bricolage ability) between X and Y. The mentioned Model Nos. 4 and 15 are shown in Figure 3.

## Findings

### The Respondents' Background

The result of the analysis of the demographic variables of the respondents is shown in Table 2. The majority of respondents were female (74.6%), ranging in age from 41 to 50 years (44.8%). The majority of these received master's degrees (53.8%) and were employed in companies that had a workforce of more than 501 workers (50.7%). A slight majority had experience in entrepreneurship ranging from 21 to 30 years. A total of 15% of the respondents were located in Shanghai.

**Table 2:** Descriptive summary of sample distribution analysis (*n* = 692).

**Demographic variables**	**Groups**	***n***	**Percentage**
Gender	Female	516	74.6
	Male	176	25.4
Age	Younger than 30 years old	73	10.5
	31–40 years old	181	26.2
	41–50 years old	310	44.8
	Older than 51 years old	128	18.5
Education	Some high school or under	132	19.1
	Bachelor degree	172	24.9
	Master degree	372	53.8
	Doctoral degree	16	2.3
Company Size	Less than 30 works	156	22.5
	31–50 workers	70	10.1
	101–500 workers	15	2.2
	Over 501 workers	351	50.7
Seniority	Less than 10 years	195	28.2
	11–20 years	214	30.9
	21–30 years	216	31.2
	Higher than 31 years	67	9.7
Location	Beijing	55	7.9
	Chongqing	27	3.9
	Fujian Province	48	6.9
	Hainan	14	2.0
	Hebei Province	21	3.0
	Henan Province	7	1.0
	Hubei Province	14	2.0
	Hunan Province	14	2.0
	Jiangsu Province	97	14.0
	Jiangxi Province	7	1.0
	Shanghai	104	15.0
	Zhejiang Province	76	11.0
	Guangzhou City	90	13.0
	Shenzhen	82	11.8
	Taiwan	36	5.2

*Source: By author*.

### Validity and Reliability Check

#### Constructive Validity Test

The survey consisted of 27 items in total. The constructive validity test results are shown in Table 3. The factor loading value ranged from 0.576 to 0.882 and this consisted of the values for the original factor and dimension. A standard *T*-value of greater than 1.96 was used in the calculation. The results significantly supported the factor loading. Meanwhile, the Kaiser-Meyer-Olkin value is 0.888 and the Bartlett test result χ^2^ is 11379.779 (*p* < 0.001), which appropriately supported the exploratory factor analysis, and the coefficient of “total variance explanation” was 68.814%.

**Table 3:** The exploration factor analysis of the developed instrument (*n* = 692).

**Factors**	**Items**	**Factor loading**	***T*-Value^a^**
Entrepreneurial	EIpa3	0.787	26.159^***^
Intention: Proactivity	EIpa1	0.684	22.557^***^
	EIpa2	0.576	20.462^***^
	EIar1	0.813	9.073^***^
Risk taking	EIar2	0.811	7.719^***^
	EIar3	0.692	6.872^***^
	EIino2	0.882	6.156^***^
Innovativeness	EIino1	0.839	5.715^***^
	EIino3	0.824	4.476^***^
Social capital	SC4	0.802	16.176^***^
	SC2	0.799	16.171^***^
	SC3	0.765	15.930^***^
	SC5	0.705	14.998^***^
	SC1	0.697	13.972^***^
Resource bricolage	RB2	0.858	15.672^***^
	RB3	0.854	15.657^***^
	RB4	0.846	15.125^***^
	RB7	0.804	13.969^***^
	RB6	0.780	13.466^***^
	RB5	0.763	14.364^***^
	RB1	0.712	10.941^***^
	RB8	0.608	8.547^***^
Entrepreneurial attitude	EA3	0.872	35.666^***^
	EA2	0.854	29.521^***^
	EA4	0.832	25.388^***^
	EA5	0.820	17.818^***^
	EA1	0.653	13.689^***^

a *Used the formula (|O/STDEV|) >1.96*.

*** *p < 0.001*.

*Source: By author*.

The analysis of discrimination validity adopted the HTMT (heterotrait–monotrait ratio of correlations) method, that is, the confidence interval (C.I.) does not include 0.0 (Voorhees et al., 2016). The results show that the C.I. coefficients range from 0.1.870 to 0.723, and none of them had a value of 0.0, which shows that the instrument has discrimination validity.

#### Reliability Test

The Cronbach's α coefficients for each variable ranged from 0.779 to 0.918, all of which were greater than the acceptable standard value of more than 0.50 (Schweizer, 2011; Adamson and Prion, 2013). The values of composite reliability (CR value) ranged from 0.707 to 0.948, which were higher than the standard value of more than 0.70 (Bagozzi and Yi, 1988; Peterson and Kim, 2013; Padilla and Divers, 2016). The calculated result of the ‘average variation extraction' (AVE) ranged from 0.517 to 0.715, which was higher than the standard value of 0.50 (Costea et al., 2017), see (Table 4). It demonstrated the reliability of the scale was supported by the statistics.

**Table 4:** The Items and dimensions of the developed questionnaire.

**Factors**	**Dimensions**	**Items**	**Cronbach's α**	**CR (ρc)**	**AVE**
Entrepreneurial	Proactivity	3	0.779	0.716	0.679
Intention	Risk taking	3	0.785	0.707	0.715
	Innovativeness	3	0.880	0.782	0.703
Social capital	–	5	0.845	0.823	0.563
Resource bricolage	–	8	0.918	0.936	0.528
Entrepreneurial attitude	–	5	0.909	0.948	0.517

*Source: By author*.

### The Estimation of Path Coefficients

Using the independent variable “social capital” (X) to predict the dependent variable “entrepreneurial attitude” (M) (β = 0.246, *p* < 0.001) and “entrepreneurial intention” (Y) (β = 0.471 *p* < 0.001) reached the level of significance. The ability of “entrepreneurial attitude” (M) to predict “entrepreneurial intention” (Y) (β = 0.281, *p* < 0.001) was significant as well. Meanwhile, the mediating effects are to be verified by the Sobal Test (Preacher and Hayes, 2008). The result shows that the Sobal Coefficient was 6.186 (*p* < 0.001), reaching a significant level. Thus, it supports the factor that “entrepreneurial attitude” has the mediating effect between “social capital” and “entrepreneurial intention.”

**Figure 4:**
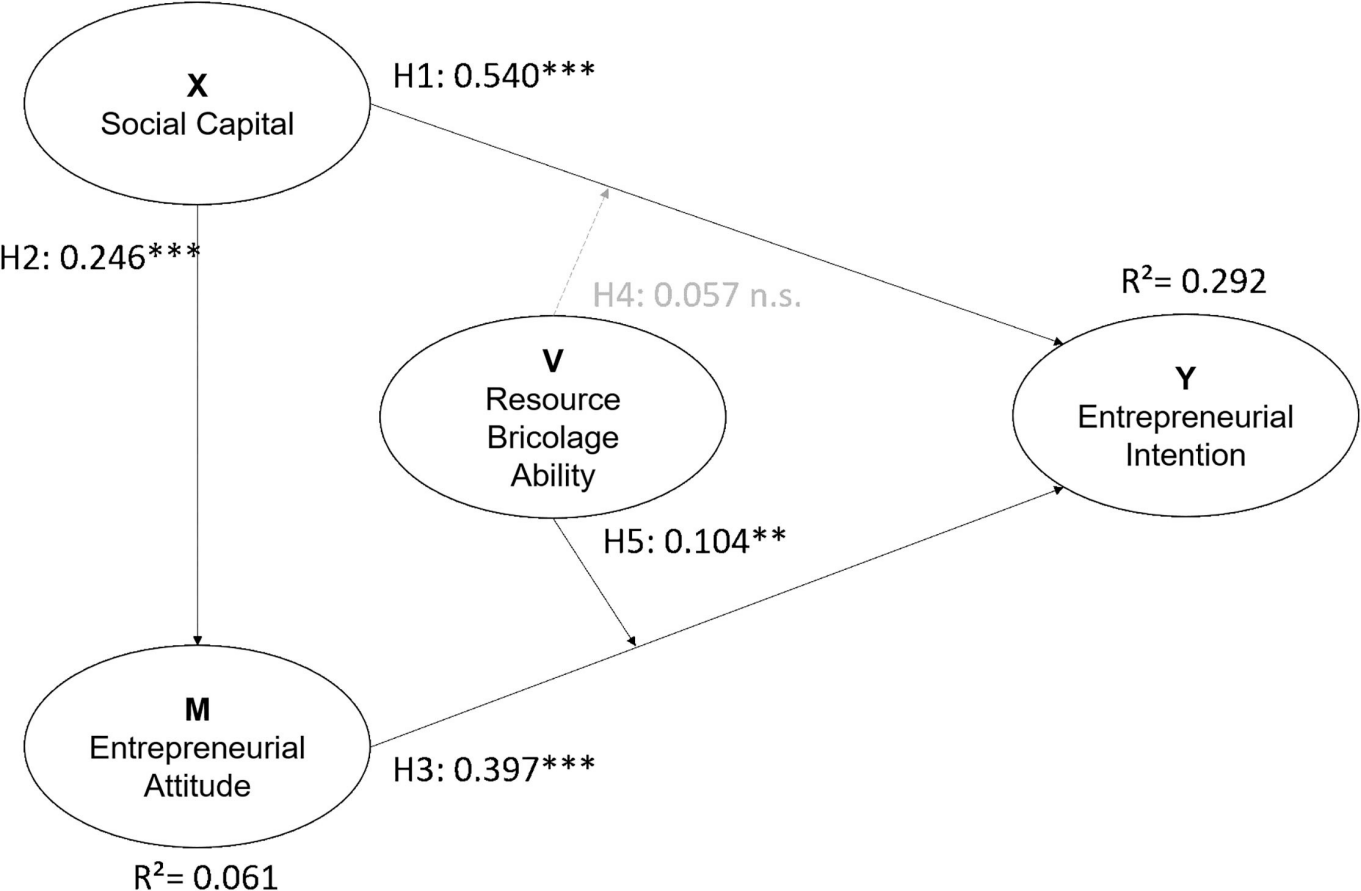
The hypotheses model with standardized path coefficient tested results.

Furthermore, the “resource bricolage ability” (V) demonstrated a moderating effect. Results showed this moderating effect did not appear between “social capital” (X) and “entrepreneurial intention” (Y) (β = 0.057, *p* > 0.05), but it did have of moderating effect between “entrepreneurial attitude” (M) and “entrepreneurial intention” (Y) (β = 0.104, *p* < 0.01); the estimated coefficients are summarized in Table 5, and the path coefficients are shown in Figure 4.

**Table 5:** The moderating effect estimated result summary (*n* = 692).

**Independent variable X** **(Social capital)**		**Dependent variable Y** **(Entrepreneurial intention)**				
**Variables**		***b***	***SE***	**β**	**T**	***p***
Constant	*i_*Y*_*	4.240	0.015	—	274.808	<0.001
XY _(SCtoEI)_	*C_1_'*	0.540	0.032	0.540	16.871	<0.001
XM _(SCtoEA−)_	*a_*i*_*	0.001	0.037	0.246	6.678	<0.001
V _(RBtoEI)_	*C_2_'*	0.130	0.019	0.257	6.994	<0.001
V_1(SC*RBtoEI)_	$C_{3}^{\prime }$	0.029	0.016	0.057	1.787	>0.050
V_2__(EA*RBtoEI)_	*b_1*i*_*	0.049	0.017	0.104	2.811	<0.010
MY _(EAtoEI)_	*b_2*i*_*	0.397	0.035	0.397	11.373	<0.001
*F*_(3,688)_ = 117.618, *p* < 0.001		*R*^2^ = 0.292				

*SC, Social Capital; EA, Entrepreneurial Attitude; RB, Resource Bricolage; EI, Entrepreneurial Intention*.

*Source: By author*.

**Figure 5:**
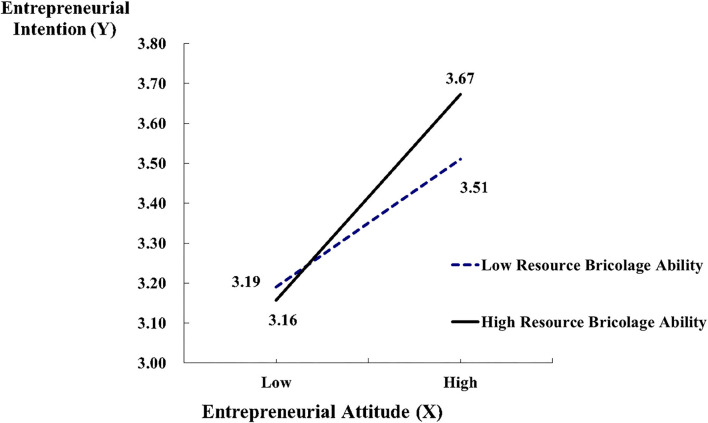
The moderating effect interaction results.

According to the moderating effect, the result of the interaction is shown in Figure 5. It demonstrated that the high-resource bricolage ability group performed slightly lower in entrepreneurial attitude (*M* = 3.16) than the low-resource bricolage ability group (*M* = 3.19). The results were reversed concerning the entrepreneurial intention score. The former group scored 3.76 while the latter scored 3.51.

Therefore, it demonstrates that the resource bricolage ability plays an important moderating role between entrepreneurial attitude and entrepreneurial intention.

## Conclusion

### Summary

Society has been growing dramatically and digital human resource development has not followed this trend sufficiently. Furthermore, the result of this study is required to meet the perspective of the psychology of high performance. It demonstrated the mental model and quality of entrepreneurs and their behaviors significantly. Furthermore, social and economic development relies on innovation and creativity by cultivating people's entrepreneurial attitudes and intentions. However, related studies did not fully consider the resource bricolage ability of entrepreneurs and its impact on entrepreneurial attitude and entrepreneurial intention, especially its moderating effect. Thus, this research provides a hypothesized model to explore the relationships among social capital, resource bricolage ability, entrepreneurial attitude, and entrepreneurial intention.

The invited participants were required to answer the designed questionnaires from China and Taiwan. The respondents were mainly females with graduate education working at enterprises with over 501 employees or fewer than 30 workers. The seniority of most ranged from 11 to 30 years. This is a reflection of their rich entrepreneurial experience. This instrument demonstrated good reliability and validity concerning the research variables. Through regression analysis, it was found that social capital could predict entrepreneurial attitude and entrepreneurial intention. Meanwhile, entrepreneurial attitude can directly impact entrepreneurial intention as well. Examination of the moderating effect of resource bricolage ability on the relationship between social capital and entrepreneurial intention and on the relationship between entrepreneurial attitude and the entrepreneurial intention was demonstrated. The moral of this result reflected that an entrepreneur who enriches his social capital would influence his entrepreneurial attitude directly and significantly. This result echoes Mahfud et al. (2020) findings. Furthermore, social capital could affect an individual's entrepreneurial intention positively and significantly as well. This result is the same as Lan and Luc (2020) and Ridha and Wahyu (2017) argument. In addition, the individual's entrepreneurial attitude could mediate social capital and entrepreneurial intention, which means social capital would not solely affect his or her entrepreneurial intention directly, the entrepreneurial attitude plays a critical role in these factors in the practical working environment. Therefore, enhancing entrepreneurs' entrepreneurial intentions must facilitate their entrepreneurial attitude. Of course, social capital was the priority to trigger this mechanism. However, research has shown that resource bricolage ability does not play a moderating role between social capital and entrepreneurial intention.

Furthermore, regarding the management style and enterprise culture differences between China and Taiwan. This study was focused on exploring the mindset of the selected samples; however, it was found from data analysis that the mindsets of these subjects were at the same level and independent of the discrepancy in management style and enterprise culture. Thus, it supported that the entrepreneurs' mental qualities were similar.

In addition, resource bricolage ability plays a moderating role between entrepreneurial attitude and entrepreneurial intention. The most important thing is that the high-resource bricolage ability group with a high entrepreneurial attitude will show higher entrepreneurial intention as compared to the low-resource bricolage ability group dramatically. Thus, it demonstrated that an individual's resource bricolage ability is the key factor to leverage the entrepreneurial attitude and entrepreneurial intention. Therefore, based on the digital human resource development perspective in the technology environment, it requires a training program for entrepreneurial ability that should provide for people to have the ability of problem-solving by using their resource bricolage ability and facilitate their new business or running a business to success.

### Suggestions

#### For Entrepreneurs

To keep successful entrepreneurial career development, it is suggested that the bricolage was a very important ability to support the business ongoing and overcome the unexpected challenges, e.g., COVID-19 or economic change.

#### For Individuals Who Tend to Entrepreneurship

As per research findings, cultivating rich social capital could promote the proportion of startups successfully. However, the bricolage ability still plays an essential role to affect the entrepreneurial intention significantly between entrepreneurial attitudes. Indeed, the cultivation of problem-solving ability will lead individuals to higher performance in startups as well.

#### For Researchers

It was suggested that future researchers could involve more samples to test the research model and extend wider factors as study variables to explore more relationships among the research model of this study.

### Strengths of This Study

The strengths of this study are advanced and ahead of the trend of social capital, entrepreneurial attitude, resource bricolage ability, and entrepreneurial intention-related studies. Furthermore, this study constructed a reliable instrument to measure the proposed factors and the results are meaningful valuable contributions to the human resource development and entrepreneurial education studies. Moreover, per research findings, the entrepreneurs should review their own resource bricolage ability. Furthermore, checking the resource bricolage ability may trigger their entrepreneurial attitude and intention, especially in the Chinese cultural context. As Chinese people have the common characteristic of diligence, active, interpersonal-relationship centered, and perseverance (Yoo, 2020), therefore, the research finding reflected these essential traits. In addition, it supported that an individual who has high mental quality will cause high performance and will be positively associated with his resource bricolage, social capital, entrepreneurial attitude, and intention.

### Limitations

The limitations of this study are: (1) the age scale of the participants was comparatively larger, which will affect the analysis results due to the cognition and awareness being different between those samples of different ages. (2) The samples were selected from Asia society with the Chinese cultural context, thus, the results are not suitable for induction worldwide due to the interaction between entrepreneurial environment and motivation being different from the western society. (3) The subjects were a unique group and they served as managers, executives, or owners of enterprises. Thus, these subjects were suited to answer the survey questions depending on their particular experience and social status. Therefore, the self-selection bias will limit the inference of this study.

## Data Availability Statement

The original contributions presented in the study are included in the article/supplementary material, further inquiries can be directed to the corresponding authors.

## Ethics Statement

The studies involving human participants were reviewed and approved by Yuanpei University of Medical Technology, Hsinchu, Taiwan. The patients/participants provided their written informed consent to participate in this study.

## Author Contributions

Y-HY is contributed to all the work of this study, which includes the research plan, conduct, survey design, data collection, data analysis, and report writing.

## Funding

This study was funded by the Faculty Research Grant Support Project of Chinese Culture University in 2021.

## Conflict of Interest

The author declares that the research was conducted in the absence of any commercial or financial relationships that could be construed as a potential conflict of interest.

## Publisher's Note

All claims expressed in this article are solely those of the authors and do not necessarily represent those of their affiliated organizations, or those of the publisher, the editors and the reviewers. Any product that may be evaluated in this article, or claim that may be made by its manufacturer, is not guaranteed or endorsed by the publisher.
